# Site-specific HNK-1 epitope on alternatively spliced fibronectin type-III repeats in tenascin-C promotes neurite outgrowth of hippocampal neurons through contactin-1

**DOI:** 10.1371/journal.pone.0210193

**Published:** 2019-01-10

**Authors:** Ayasa Nakamura, Jyoji Morise, Keiko Yabuno-Nakagawa, Yuki Hashimoto, Hiromu Takematsu, Shogo Oka

**Affiliations:** Department of Biological Chemistry, Human Health Sciences, Graduate School of Medicine, Kyoto University, Kyoto, Japan; University of Louisville, UNITED STATES

## Abstract

The human natural killer-1 (HNK-1) carbohydrate epitope, composed of a unique sulfated trisaccharide (HSO_3_–3GlcAβ1–3Galβ1–4GlcNAc-R), is highly expressed during brain development and regulates higher brain function. However, it remains unclear which glycoprotein carries the HNK-1 epitope in the embryonic brain and the functional role it plays. Here, we showed that one of the major HNK-1 carrier proteins in the embryonic brain is tenascin-C (TNC), an extracellular matrix protein that regulates neurite outgrowth by interacting with the GPI-anchored protein contactin-1 (CNTN). Because the alternatively spliced fibronectin type-III (FNIII) repeats in TNC give rise to many isoforms and affect neuronal function, we evaluated neurite outgrowth of primary hippocampal neurons on purified recombinant FNIII repeats with or without the HNK-1 epitope as a substrate. We found that the presence of the HNK-1 epitope on the C domain of TNC promoted neurite outgrowth, and that this signal was mediated by CNTN, which is an HNK-1-expressing neuronal receptor. The neurite-promoting activity of the HNK-1 epitope on TNC required neuronal HNK-1 expression, which was defective in neurons lacking the glucuronyltransferases GlcAT-P and GlcAT-S. These results suggest that the HNK-1 epitope is a key modifier of TNC and CNTN in the regulation of embryonic brain development.

## Introduction

The human natural killer-1 (HNK-1) carbohydrate epitope is a trisaccharide moiety with a sulfated glucuronic acid at the nonreducing terminal (HSO_3–_3GlcAβ1–3Galβ1–4GlcNAc-R), and is highly expressed in the nervous system [1]. This epitope was synthesized in the Golgi apparatus by a tandem reaction of one of two glucuronyltransferases, GlcAT-P or GlcAT-S, and the sulfotransferase HNK-1ST [2–4]. Of the two glucuronyltransferases, GlcAT-P is considered the key enzyme for HNK-1 expression in the brain, because GlcAT-P-deficient mice displayed almost complete loss of the HNK-1 epitope in the brain. Moreover, GlcAT-P-deficient mice exhibited impaired memory and learning with substantial reduction of long-term potentiation (LTP) in the adult hippocampal CA1 region [5], which indicates that the HNK-1 epitope is important for higher brain function. HNK-1 expression peaks at an early postnatal stage and gradually decreases in adulthood [6]. The HNK-1 epitope is only expressed on limited glycoproteins involved in neuronal recognition and synaptic plasticity (e.g., Myelin protein zero [MPZ], NCAM, tenascin-R, and GluA2) [6–9]. Importantly, expression of the glycan epitope is regulated independently from the expression of carrier proteins. For example, MPZ, which possesses only a single potential *N*-glycosylation site, can be present with or without the HNK-1 epitope in peripheral myelin [10]. Therefore, the neuronal functions of these carrier proteins might depend on the spatiotemporal expression of the HNK-1 epitope or other modifications. However, the HNK-1 carrier glycoprotein and the role of the HNK-1 epitope in the embryonic brain have not been elucidated.

Tenascin-C (TNC) is an extracellular matrix protein that is highly expressed in early brain development and is involved in neurite outgrowth and axon guidance [11]. Human TNC contains repeats of fibronectin type-III (FNIII) domains that are composed of eight constitutive (numbered 1–8) and nine alternatively spliced FNIII repeats (named A1, A2, A3, A4, B, AD2, AD1, C, and D domains), which are inserted between the fifth and sixth constitutive FNIII repeats [12]. Various alternative splice patterns give rise to the different isoforms and thus different functions of TNC. Among the alternatively spliced FNIII repeats, the B and D domains significantly promote neurite outgrowth through contactin-1 (CNTN), which activates phospholipase C (PLC)-dependent pathways [13]. A recent report showed that TNC is a major HNK-1 carrier protein in embryonic neural stem cells prepared from the striata of mouse embryos, and that the presence of the HNK-1 epitope on TNC is involved in the proliferation of neural stem cells [14]. However, it is unclear whether TNC is the major HNK-1 carrier protein in the embryonic brain, and if so, whether the HNK-1 epitope on TNC affects its neurite-promoting activity.

In this study, we confirmed that the HNK-1 epitope was primarily expressed on TNC in the embryonic day 16 (E16) mouse brain, and that its expression was nearly abolished in developmental stages after postnatal day 7 (P7). The neurite-promoting activity of TNC was enhanced by expression of the HNK-1 epitope. However, the effects of the HNK-1 epitope were suppressed following treatment with an anti-CNTN antibody, which indicates that CNTN mediates neurite outgrowth induced by the HNK-1 epitope on TNC. Furthermore, the neurite-promoting activity of the HNK-1 epitope on TNC was not observed in neurons lacking GlcAT-P and GlcAT-S, which suggests that the HNK-1 epitope on CNTN may effectively regulate neurite outgrowth. These results indicate the possibility that the HNK-1 epitope promotes TNC-derived neurite outgrowth via CNTN in the embryonic brain.

## Materials and methods

### Ethics statement

All animal experiments were conducted according to the Fundamental Guidelines for Proper Conduct of Animal Experiments and Related Activities in Academic Research Institutions under the jurisdiction of the Ministry of Education, Culture, Sports, Science and Technology of Japan and approved by the Committees on Animal Experimentation of Kyoto University. For preparation of brains and primary culture, mice were euthanized by intraperitoneal injection of sodium pentobarbital.

### Preparation of the soluble fraction of mouse brains

Whole brains dissected from E16, P0, P7, and 2- and 10-week-old (2W and 10W, respectively) mice (C57/BL6j; purchased from Shimizu Laboratory Supplies [Kyoto, Japan]) were homogenized with four volumes of homogenizing buffer (20 mM Tris-HCl [pH 7.4], 150 mM NaCl, 1 mM EDTA, and protease inhibitors [Cat# 25955–11, Nacalai Tesque, Kyoto, Japan]). The homogenates were centrifuged at 1000 × *g* for 10 min to remove nuclei, followed by centrifugation at 105,000 × *g* for 1 h. The supernatants were designated Tris-buffered saline-soluble fractions, and the pellets were re-suspended with 1% sodium dodecyl sulfate (SDS). GlcAT-P and GlcAT-S double gene-deficient mice were generated by crossing GlcAT-P and GlcAT-S gene-deficient mice [5,15].

### Immunoprecipitation and glycosidase digestion

For immunoprecipitation, primary antibodies (20 μg/mL) and protein G-Sepharose 4 Fast Flow (Cat# 17061801, GE Healthcare, Chicago, IL, USA) were added to the soluble fractions and rotated at 4°C for 2 h. Following centrifugation at 3000 × *g* for 2 min, the supernatant was obtained as an unbound fraction. Precipitated proteins were eluted with 1% SDS, giving rise to the bound fraction. For glycosidase treatment, proteins of the soluble fraction of the mouse brain were precipitated with ethanol, re-suspended, and denatured with 0.5% SDS at 100°C for 5 min. Then, to reduce the concentration of SDS, the solution was diluted with four volumes of phosphate-buffered saline (PBS) containing 4 mM EDTA, 0.5% NP-40, and 1% 2-mercaptoethanol (final concentrations). Peptide *N*-glycosidase F (PNGase F; Cat# 11365177001, Roche Applied Science, Basel Switzerland) was added at a final concentration of 20 mU/μL, followed by incubation at 37°C for 16 h.

### Expression plasmids

The HNK-1 expression plasmid, GlcAT-P-IRES-HNK-1ST/pIRES, was previously generated [16]. The human full-length TNC expression plasmid, TNC-Halo7/pFN21A, was obtained from Promega Corporation (Cat# FHC00319, Durham, NC, USA). FNIIIx7-myc-His/pCDNA3.1B was constructed as follows. First, alternatively spliced FNIII repeats (composed of A1, A2, A3, A4, B, C, and D domains) were amplified from TNC-Halo7/pFC14 using the primer pair 5’-TTTGGATCCGAACAAGCCCCTGAGCTGG-3’ and 5’-GGGTCTAGAGCTGTTGTTGCTATAGCACTGAC-3’, cloned into the *BamH*I and *Xba*I sites of pCDNA3.1, and inserted in-frame with the myc-His sequence of pCDNA3.1B (Cat# V80020, Invitrogen). To introduce the signal peptide derived from TNC, the signal peptide sequence in TNC-Halo7/pFC14 was amplified using the primer pair 5’-TTTGGATCCATGGGGGCCATGACTCAGCTG-3’ and 5’-TTTGGATCCACCTTCGGTAGCGAGGGCAAG-3’ and cloned into the *BamH*I site, resulting in FNIIIx7-myc-His/pCDNA3.1B. To construct FNIIIx6-myc-His, FNIIIx5-myc-His, FNIIIx4-myc-His, and FNIIIx3-myc-His/pCDNA3.1B, corresponding sequences were amplified from FNIIIx7-myc-His/pCDNA3.1B using the following primer pairs: 5’-TTTAAGCTTATGGGGGCCATGACTCAGCTG-3’ and 5’-GGGTCTAGAGCTGTAACAATCTCAGCCCTC-3’ (for FNIIIx6-myc-His), 5’-GGGTCTAGAGCTGTCGTGGCTGTGGCACTG-3’ (for FNIIIx5-myc-His), 5’-GGGTCTAGAGCTGTGGAGGCCTCAGCAGAG-3’ (for FNIIIx4-myc-His), or 5’-GGGTCTAGAGCTGTGACGACCTCTACAGC-3’ (for FNIIIx3-myc-His), cloned into the *Hind*III and *Xba*I sites of pCDNA3.1, and introduced in-frame with the myc-His sequence of pCDNA3.1. FNIIIx7N1534Q-myc-His/pCDNA3.1B was constructed using the QuikChange Lightning Site-Directed Mutagenesis Kit (Cat# 210518, Stratagene, San Diego, CA, USA) with the following primers: 5’-CTGCCCCTTCTGGAACAGCTAACCATTTCCGAC-3’ and 5’-GTCGGAAATGGTTAGCTGTTCCAGAAGGGGCAG-3’.

### Cell culture and transfection

HEK293 cells purchased from the American Type Culture Collection (ATCC, Manassas, VA, USA) were cultured in Dulbecco’s Modified Eagle Medium (Cat# 08456–65, Nacalai Tesque) supplemented with 10% (v/v) fetal bovine serum (Cat# 172012, Sigma-Aldrich, St. Louis, MO, USA). The HEK293 cell line was authenticated by PowerPlex16 STR (contract to Promega). Cells were transfected in Opti-MEM (Cat# 31985–070, Gibco, Gaithersburg, MD, USA) with cDNAs using Polyethylenimine “Max” (Cat# 24765–1, Polysciences, Warrington, PA, USA) according to the manufacturer’s instructions. After 6 h, the Opti-MEM medium was replaced with ASF medium 104 (Cat# 104, Ajinomoto, Tokyo, Japan) and the medium was collected after 72 h. Primary hippocampal neurons were prepared from E16 mouse brains as follows. Hippocampi were trypsinized at 37°C for 8 min and treated with 0.033% DNase I solution (Cat# D5052, Sigma-Aldrich) for 1 min. Then dissociated neurons were plated at 1.2 × 10^5^ cells per poly-D-lysine (PDL)-coated 4-well culture slide (Cat# 354577, Corning, Corning, NY, USA) in neurobasal medium (Cat# 21103–049, Invitrogen, Carlsbad, CA, USA) supplemented with 2% B27 (Cat# 17504–44, Invitrogen) and 500 μM L-glutamine (Cat# 16948–04, Nacalai Tesque).

### Purification of substrates

Culture medium obtained from HEK293 cells transfected with cDNAs was applied into the column packed with Ni-NTA agarose beads (Cat# 30210, Qiagen, Gaithersburg, MD, USA). Bound proteins were eluted with elution buffer (20 mM phosphate buffer [pH 8.0], 300 mM NaCl, and 300 mM imidazole). Purified substrates were coated on PDL-coated 4-well culture slides at 37°C for 24 h (10 μg/mL in Hank’s balanced salt solution).

### Glycosidase digestion of substrates

Culture medium obtained from HEK293 cells transfected with cDNAs was rotate with Ni-NTA agarose beads at 4°C for 16 h. Bound proteins were eluted with 1% SDS at 100°C for 5 min. Then, to reduce the concentration of SDS, the solution was diluted with four volumes of PBS containing 4 mM EDTA, 0.5% NP-40, and 1% 2-mercaptoethanol (final concentrations). Then, PNGase F was added at a final concentration of 20 mU/μL, followed by incubation at 37°C for 16 h.

### SDS-PAGE, immunoblotting, and protein staining

Soluble fractions from mouse brains, immunoprecipitates, or purified proteins were separated using 7% or 10% SDS-polyacrylamide gel electrophoresis (SDS-PAGE) and transferred to nitrocellulose membranes. For molecular size marker, Precision Plus Protein All Blue Prestained Protein Standards were used (Cat#1610373, Bio-Rad, Hercules, CA, USA). After blocking in 5% nonfat dried milk in PBS containing 0.05% Tween-20, the membranes were incubated with specific primary antibodies (1 μg/mL) followed by appropriate horseradish peroxidase (HRP)-conjugated secondary antibodies (1 μg/mL). Then proteins were detected with SuperSignal West Pico Chemiluminescent Substrate (Cat# 34580, Thermo Fisher Scientific, Waltham, MA, USA) using the Luminoimage Analyzer LAS-3000 (FujiPhoto Film, Tokyo, Japan). The following antibodies were used in the immunoblotting or immunoprecipitation experiments: mouse HNK-1 monoclonal (a hybridoma cell line was purchased from ATCC [AB_10013722]), rat anti-TNC monoclonal (T3413 [AB_477574]; Sigma-Aldrich), mouse anti-Tubulin monoclonal (T8535 [AB_261795]; Sigma-Aldrich), rabbit anti-myc polyclonal (ab9106 [AB_307014]; Abcam, Cambridge, MA, USA), and goat anti-contactin-1 polyclonal (GT15055 [AB_2737187]; Neuromics, Edina, MN, USA) primary antibodies; and HRP-conjugated goat anti-mouse IgM, rabbit anti-rat IgG, goat anti-rabbit IgG, and rabbit anti-goat IgG (62–6820 [AB_2533954], 61–9520 [AB_2533945], 65–6120 [AB_2533967], and 81–1620 [AB_2534006], respectively; Invitrogen) secondary antibodies. For protein staining, polyacrylamide gels were stained with Coomassie Brilliant Blue (CBB) using GelCode Blue Stain Reagent according to the manufacturer’s instructions (Cat# 24590, Thermo Fisher Scientific). Bovine serum albumin (BSA; Cat# 01860–07, Nacalai Tesque) was applied as a loading control.

### Immunostaining and evaluation of neurite outgrowth

At day 3 *in vitro* (DIV3), hippocampal primary neurons were fixed in methanol at -20°C for 20 min and incubated with mouse anti-Tau-1 monoclonal antibody (mAb) (5 μg/mL, MAB3420 [AB_94855]; Millipore, Stafford, VA, USA) in PBS containing 3% BSA for 1 h at room temperature, followed by incubation with Alexa Fluor 546-conjugated donkey anti-mouse IgG antibody (5 μg/mL, A10036 [AB_2534012]; Molecular Probes, Eugene, OR, USA) in PBS containing 3% BSA at room temperature for 1 h. For the application of antibodies in culture medium, 30 μg/mL control goat IgG (sc-2028 [AB_737167]; Santa Cruz Biotechnology, Dallas, TX, USA) and goat anti-contactin-1 polyclonal antibody (pAb) were added after 18 h of culture. For application of the PLC inhibitor, 1 nM U73122 (Cat# U6756, Sigma-Aldrich) was added after 18 h of culture. Dimethyl sulfoxide (DMSO) was used as a control for adding U73122. Stained neurons were observed using the FluoView imaging system (Olympus, Tokyo, Japan). Neurite outgrowth was traced and evaluated with MetaMorph (BioLab, Inc., Conyers, GA, USA) software. One-way ANOVA with Tukey’s multiple comparison posttest was applied for statistical analyses by using OriginPro (OriginLab, MA, USA).

## Results

### TNC is a major HNK-1 carrier protein in the embryonic brain

**Fig 1:**
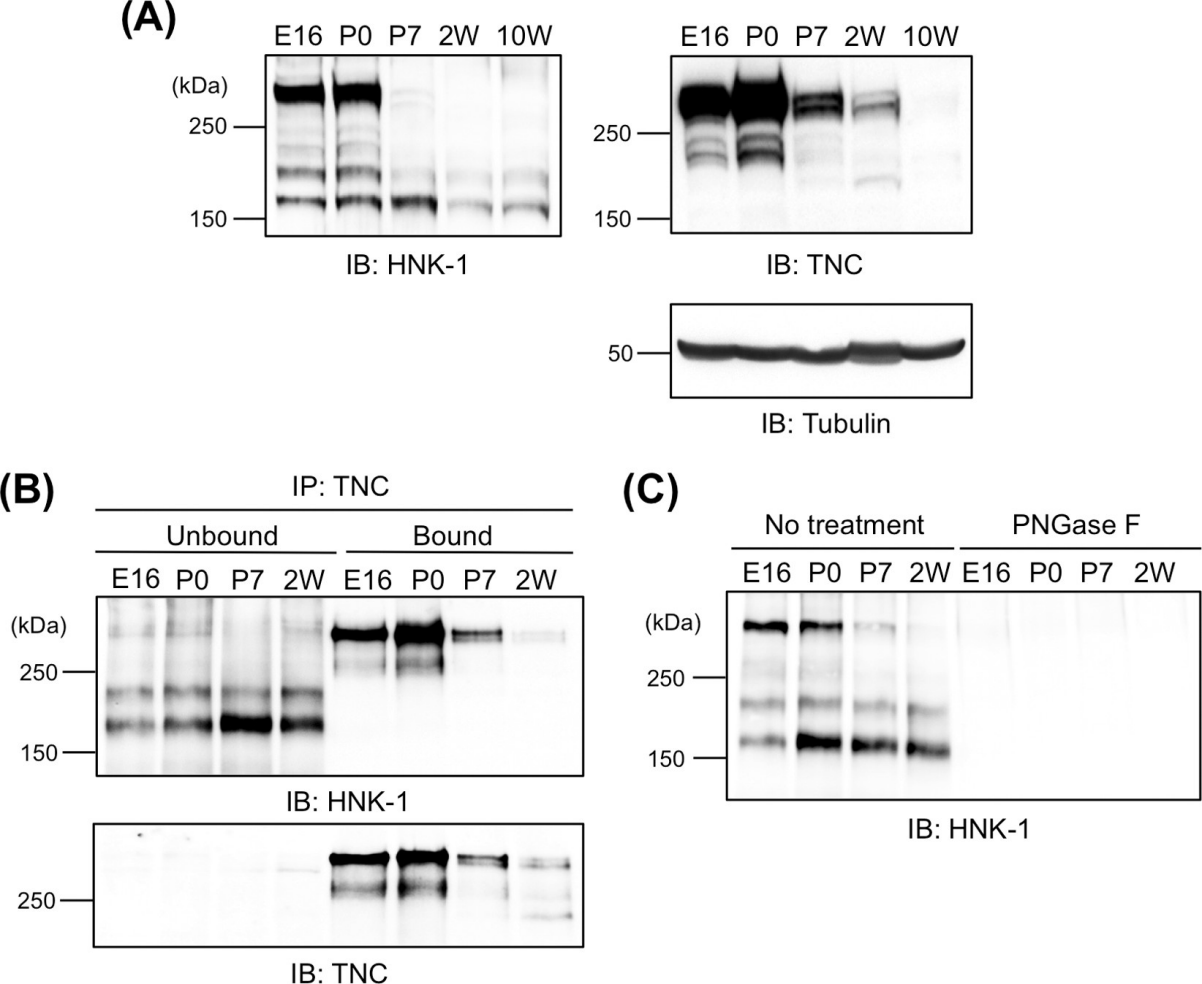
HNK-1 epitope is expressed on the *N*-glycan of tenascin-C. (A) Soluble fractions prepared from embryonic day 16 (E16), postnatal day 0 and 7 (P0 and P7, respectively), 2- and 10-week-old (2W and 10W, respectively) mouse brains were immunoblotted with HNK-1 mAb, anti-TNC pAb, and anti-Tubulin mAb (for loading control). Each blot was representative of 12 images of western blots. 50, 80, and 20 μg proteins were loaded to each lane for HNK-1, TNC, and Tubulin, respectively. (B) Soluble fractions of E16, P0, P7, and 2W mouse brains were immunoprecipitated with anti-TNC pAb and antibody-bound (Bound) and unbound (Unbound) fractions were obtained. Each blot was representative of 8 images of western blots. 200 μg proteins were used for each immunoprecipitation. (C) Soluble fractions of E16, P0, P7, and 2W mouse brains were treated with or without peptide *N*-glycosidase F (PNGase F or No treatment, respectively). The blot was representative of 5 images of western blots. 20 μg proteins were loaded to each lane.

A recent report showed that TNC is a major HNK-1 carrier protein in embryonic neural stem cells [14]. To evaluate whether TNC is also a major glycoprotein carrier in the mouse embryonic brain, soluble fractions of whole brains prepared from E16.5 to 10W were analyzed by immunoblotting with HNK-1 mAb. The majority of HNK-1 signals were detected at greater than 250 kDa at E16 and P0, and HNK-1 expression largely disappeared after P7 (**Fig 1A**, left). When immunoblotted with anti-TNC pAb, the majority of TNC signals were also greater than 250 kDa, corresponding to the band detected by the HNK-1 mAb, which suggests that TNC is a major glycoprotein carrier of the HNK-1 epitope even in the embryonic brain (**Fig 1A**, right upper). For confirmation, the soluble fractions were subjected to immunoprecipitation with anti-TNC pAb and antibody-bound and unbound fractions were obtained (**Fig 1B**). When TNC was observed in the bound fractions, > 250 kDa HNK-1-positive bands were also observed in the bound fractions and immunodepleted from the unbound fractions although other HNK-1 carriers were present. The HNK-1-positive bands disappeared following PNGase F digestion (**Fig 1C**). Taken together, our results suggest that the HNK-1 epitope in the embryonic brain is primarily present on the *N*-glycan of TNC. Multiple TNC bands were observed, which were likely due to alternative splicing of the FNIII repeats and resulted in the production of several TNC isoforms (**Fig 1A**, right upper). The majority of the HNK-1 signals were observed as higher molecular weight bands (containing all or several alternatively spliced FNIII repeats) among the TNC isoforms, which suggests that the HNK-1 epitope is present on alternatively spliced FNIII repeats of TNC, consistent with a previous report [14].

### Expression of the HNK-1 epitope on the C domain enhances neurite outgrowth

**Fig 2:**
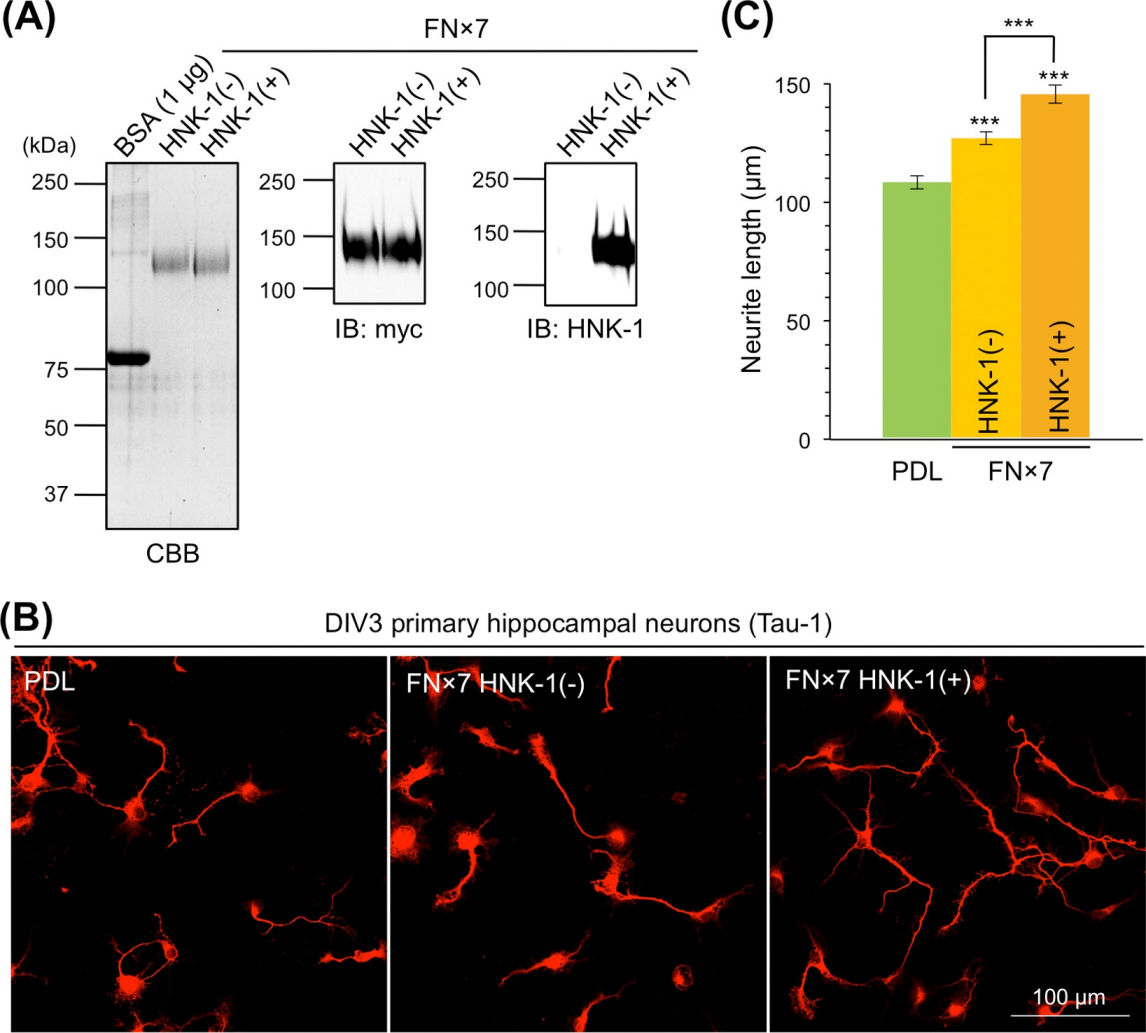
HNK-1 epitope on alternatively spliced FNIII repeats promotes neurite outgrowth. (A) Purified seven fibronectin type-III repeats (FNx7) with or without HNK-1 synthesis enzymes (HNK-1(+) or HNK-1(-), respectively) were subjected to SDS-PAGE and stained with CBB or immunoblotted with anti-myc pAb and HNK-1 mAb. Each blot was representative of 2 images of western blots. 1 and 0.2 μg proteins were loaded to each lane for CBB and western blots, respectively. (B) Primary hippocampal neurons were cultured on poly-D-lysine (PDL), PDL coated with FNx7 without HNK-1 expression (FNx7 HNK-1(-)), or PDL coated with FNx7 with HNK-1 expression (FNx7 HNK-1(+)). At day 3 *in vitro* (DIV3), neurons were immunostained with anti-Tau-1 mAb. (C) Estimation of neurite length on each coating. Green, PDL; yellow, FNx7 HNK-1(-); and orange, FNx7 HNK-1(+). Numbers of neurons measured on PDL, FNx7 HNK-1(-), and FNx7 HNK-1(+) were 63, 138, 43, respectively. This experiment was performed across seven independent cultures, and the representative result obtained from a single culture was shown. Error bars represent standard error of the mean (SEM). ***p < 0.001.

**Fig 3:**
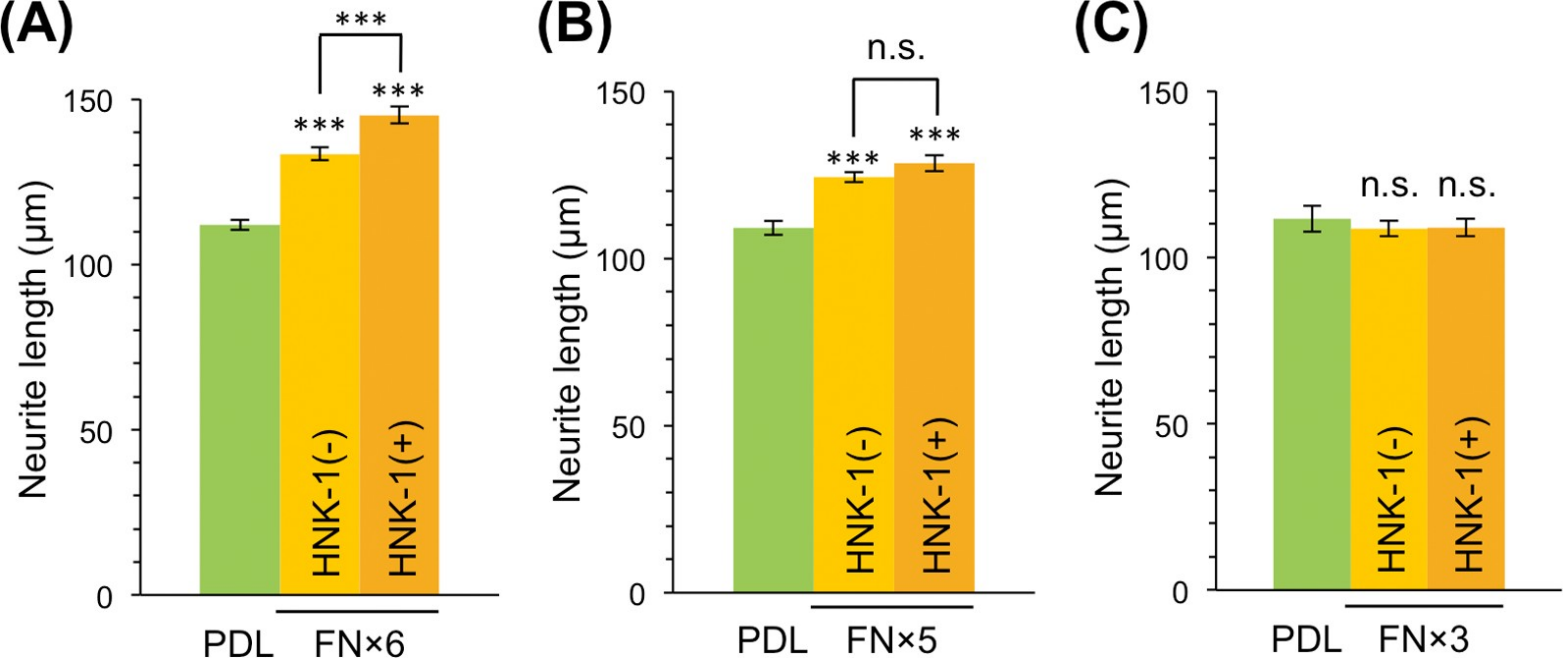
HNK-1 epitope on the C domain is effective for promoting neurite outgrowth. (A–C) Primary hippocampal neurons were cultured on PDL, PDL coated with FNx6 (A), FNx5 (B), or FNx3 (C) without HNK-1 expression (HNK-1(-)), or PDL coated with FNx6 (A), FNx5 (B), or FNx3 (C) with HNK-1 expression (HNK-1(+)). The cultured neurons were immunostained and analyzed at DIV3. Green, PDL (n = 130, 131, and 86 for A, B, and C, respectively); yellow, FNx6, FNx5, or FNx3 HNK-1(-) (n = 161, 241, and 124 for FNx6, FNx5, and FNx3 HNK-1(-), respectively); and orange, FNx6, FNx5, or FNx3 HNK-1(+) (n = 152, 143, and 92 for FNx6, FNx5, and FNx3 HNK-1(+), respectively). Numbers of measured neurons were indicated in parentheses. These experiments were performed with three independent cultures and the representative results obtained from a single culture were shown. Error bars represent SEM. ***p < 0.001; n.s. (not significant), p > 0.05.

**Fig 4:**
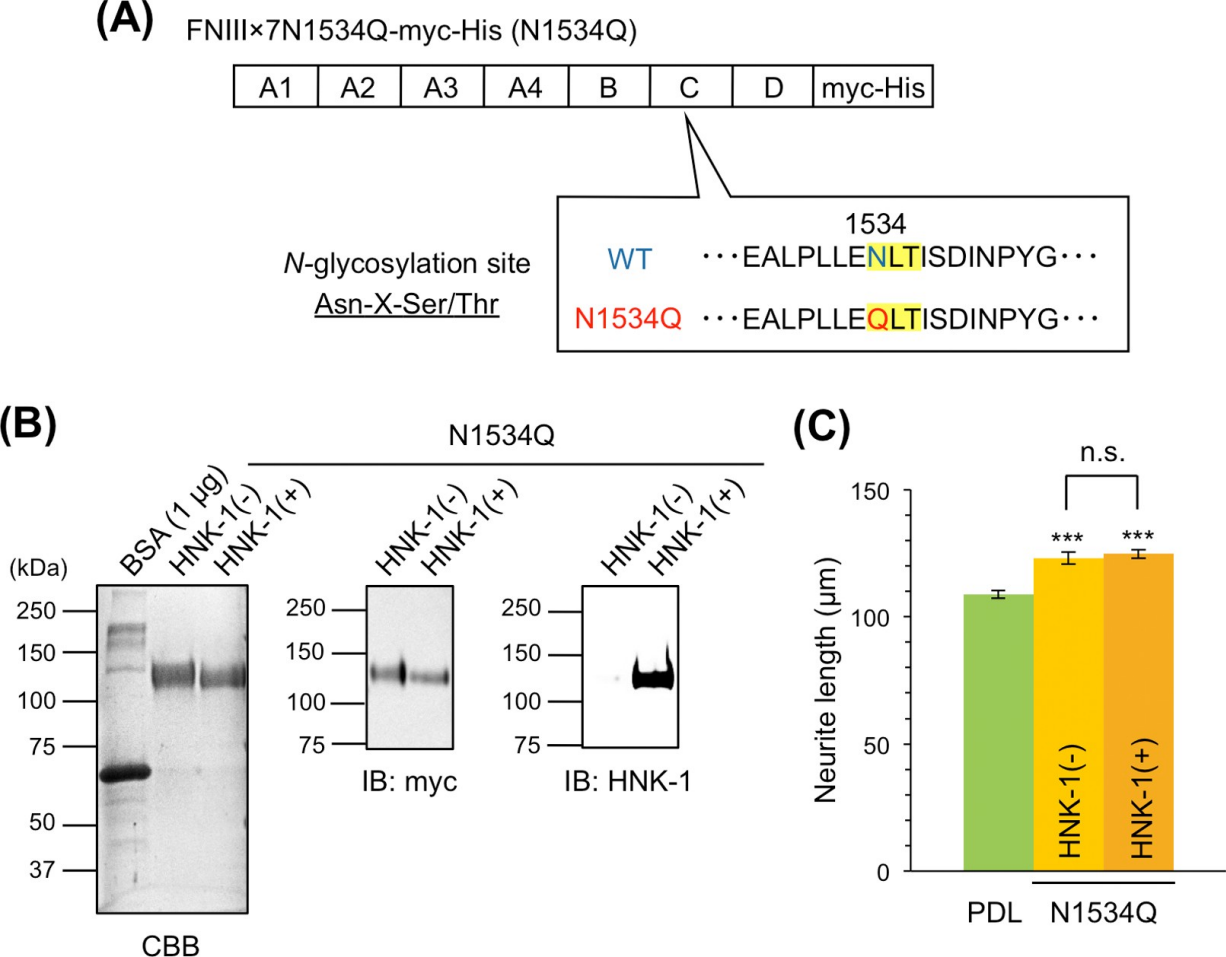
HNK-1 epitope on N1534 of the C domain affects neurite outgrowth. (A) Scheme of FNIIIx7N1534Q-myc-His (N1534Q). *N*-glycosylation site (Asn-X-Ser/Thr; highlighted in yellow) at N1534 was mutated to glutamine. (B) N1534Q was purified with or without HNK-1 synthesis enzymes (HNK-1(+) or HNK-1(-), respectively), subjected to SDS-PAGE and stained with CBB or immunoblotted with anti-myc pAb and HNK-1 mAb. Each blot was representative of 2 images of western blots. 1 and 0.2 μg proteins were loaded to each lane for CBB and western blots, respectively. (C) Primary hippocampal neurons were cultured on PDL, PDL coated with N1534Q without HNK-1 expression (HNK-1(-)), or PDL coated with N1534Q with HNK-1 expression (HNK-1(+)). Neurons were immunostained and analyzed at DIV3. Green, PDL; yellow, N1534Q HNK-1(-); and orange, N1534Q HNK-1(+). Numbers of neurons measured on PDL, N1534Q HNK-1(-), and N1534Q HNK-1(+) were 184, 197, 195, respectively. This experiment was performed across two independent cultures, and the representative result obtained from a single culture was shown. Error bars represent SEM. ***p < 0.001; n.s., p > 0.05.

The alternative splicing domains of mouse and human TNC function in neurite outgrowth and are primarily involved in the neurite-promoting activity of TNC [17–19]. However, the role of glycans in the alternative splicing domains remains unclear. Therefore, we used recombinant myc-His-tagged TNC with seven FNIII repeats (FNx7) of alternative splicing domains, which contained the A1, A2, A3, A4, B, C, and D domains (**S1A Fig**). To control HNK-1 expression, FNx7 was expressed with or without the HNK-1 synthesis enzymes, GlcAT-P and HNK-1ST, in HEK293 cells, and purified with a Ni-NTA agarose column. Purified FNx7, which was co-expressed with the HNK-1 synthesis enzymes, was clearly modified with the HNK-1 epitope (**Fig 2A**). To evaluate the neurite-promoting activity, primary hippocampal neurons were cultured on PDL with or without FNx7 and immunostained with the axonal marker Tau-1 at DIV3 (**Fig 2B**). Consistent with previous reports [13,17–19], FNx7 enhanced neurite outgrowth (**Fig 2C**). HNK-1-expressing FNx7 elongated neurite length compared to non-HNK-1 FNx7 (**Fig 2C**). Therefore, the presence of the HNK-1 epitope enhanced TNC function. However, the treatment of FNx7 with or without HNK-1 into the culture medium did not show any neurite-promoting activity, suggesting that coating the substrate might be the important method for observing this activity (**S2 Fig**). Among the alternatively spliced FNIII repeats, the B and D domains have been associated with neurite outgrowth [13,17–19], and FNx7 has 14 potential *N*-glycosylation sites (**S1A Fig**), any of which could be functional. To determine the site of the HNK-1 epitope involved in enhancing neurite-promoting activity, we generated a series of deletion constructs that deleted individual FNIII repeats from the C-terminal end (FNx6, FNx5, FNx4, and FNx3; **S1A Fig**). These constructs were expressed in HEK293 cells with or without HNK-1 synthesis enzymes. The HNK-1 epitope was present at similar levels on each recombinant protein when co-expressed with the HNK-1 synthesis enzymes, which suggests that the HNK-1 epitope is not preferentially expressed on a specific domain (**S1B Fig**). Notably, the HNK-1 epitope on each recombinant protein was completely abolished by PNGase F treatment (**S1C Fig**), indicating that the HNK-1 epitope was present only on *N*-glycans of the substrates used in this study. When primary hippocampal neurons were cultured on FNx6, the HNK-1 epitope of FNx6 enhanced the neurite-promoting activity to the same extent as FNx7 (**Fig 3A**). By contrast, although FNx5 exhibited neurite-promoting activity, which was likely due to effects of the B domain, HNK-1 expression did not facilitate neurite outgrowth (**Fig 3B**). This suggests that the HNK-1 epitope on the *N*-glycan of the C domain could be important for the enhanced neurite-promoting activity. Consistent with a previous report [18], FNx3 had no effect on neurite outgrowth (**Fig 3C**), likely because of the lack of B and D domains. These data indicate that the HNK-1 epitope on the C domain is required for enhancement of neurite-promoting activity. The C domain only has one *N*-glycosylation site (**S1A Fig**), in which an asparagine residue in the consensus sequence (N-X-S/T) of FNx7 was mutated to glutamine to generate the N1534Q mutant (**Fig 4A**). Then N1534Q was purified with or without the HNK-1 epitope (**Fig 4B**). N1534Q without the HNK-1 epitope still exhibited neurite-promoting activity as well as FNx7 (**Fig 4C**), which suggests that the lack of *N*-glycan at N1534 does not affect the function of TNC. However, HNK-1-expressing N1534Q did not exhibit enhanced neurite-promoting activity compared to non-HNK-1 N1534Q. Overall, these results suggest that the HNK-1 epitope on the *N*-glycan at the C domain plays an important role in neurite-promoting activity.

### HNK-1 mediates tenascin-C-derived neurite outgrowth via contactin-1

**Fig 5:**
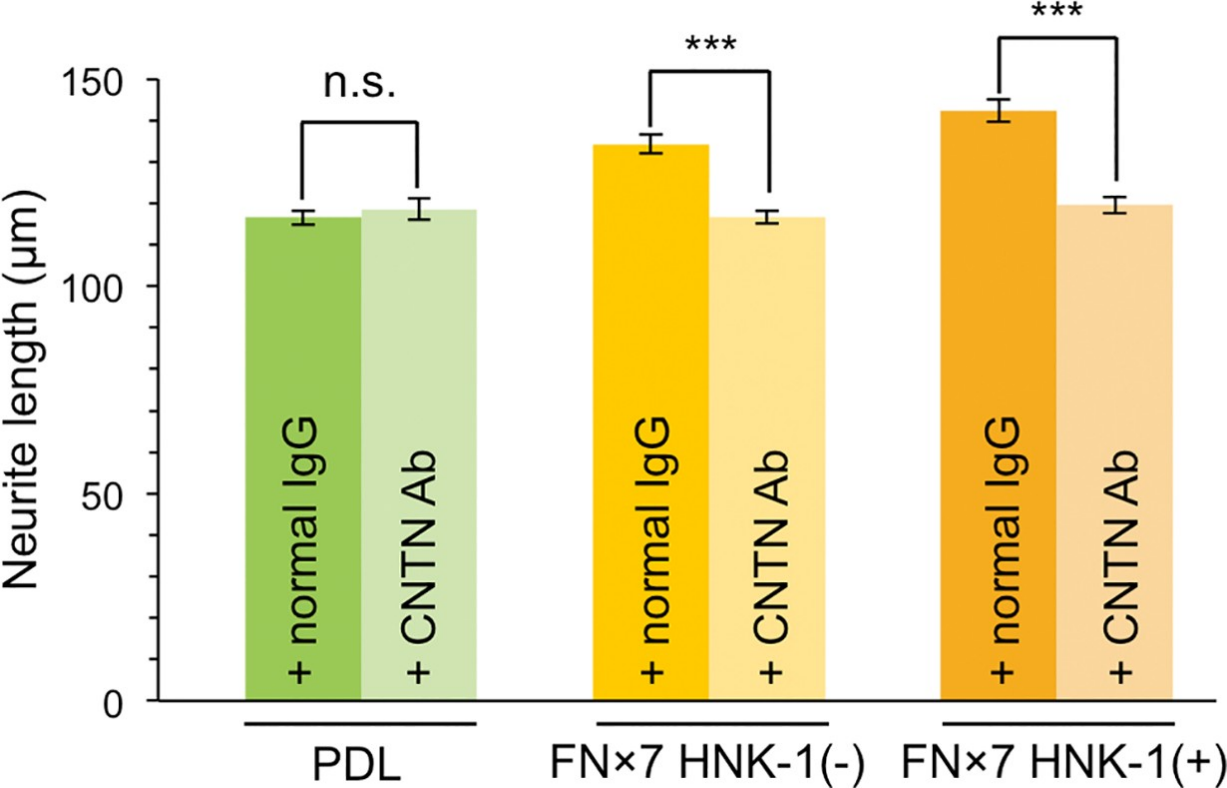
Contactin-1 mediates neurite outgrowth derived from the HNK-1 epitope on tenascin-C. Primary hippocampal neurons were cultured on PDL, PDL coated with FNx7 without HNK-1 expression (HNK-1(-)), or PDL coated with FNx7 with HNK-1 expression (HNK-1(+)). Normal goat IgG (+ normal IgG) or anti-CNTN pAb (+ CNTN Ab) was added to the culture medium at a final concentration of 30 μg/mL after 18 h of culture. Neurons were immunostained and analyzed at DIV3. Green, PDL with normal goat IgG (n = 219); light green, PDL with anti-CNTN pAb (n = 130); yellow, FNx7 HNK-1(-) with normal goat IgG (n = 139); light yellow, FNx7 HNK-1(-) with anti-CNTN pAb (n = 163); orange, FNx7 HNK-1(+) with normal goat IgG (n = 154); and light orange, FNx7 HNK-1(+) with anti-CNTN pAb (n = 166). Numbers of measured neurons were indicated in parentheses. This experiment was performed across two independent cultures, respectively, and the representative result obtained from a single culture was shown. Error bars represent SEM. ***p < 0.001; n.s., p > 0.05.

Alternatively spliced FNIII repeats interact with several counter receptors such as RPTPβ, integrin, or CNTN using various domains [20]. Among them, CNTN strongly mediates neurite outgrowth via the B and D domains [13]. To investigate whether CNTN is responsible for the HNK-1-dependent enhancement of neurite outgrowth, anti-CNTN pAb was added to the culture and neurite length, after which growth on FNx7 with or without the HNK-1 epitope was evaluated. Anti-CNTN pAb did not affect neurite outgrowth on PDL (**Fig 5**). However, adding anti-CNTN pAb suppressed neurite outgrowth on FNx7, consistent with a previous report [13]. This result suggested that anti-CNTN pAb used in this study might act as a functional inhibitor of CNTN. In these conditions, increased neurite outgrowth observed with HNK-1-expressing FNx7 was also abolished. Because neurite length on HNK-1-expressing FNx7 was comparable to that on PDL (~120 μm), CNTN may be required for the neurite-promoting activity of the HNK-1 epitope on TNC. This suggests that the HNK-1 epitope on TNC plays an additive role for TNC–CNTN signaling, but it remains unclear whether HNK-1 is directly a part of ligand for CNTN or indirectly enhances TNC–CNTN interaction.

Meanwhile, a previous report also suggested that TNC mediates activation of PLC, one of the cleavage enzymes of phosphatidylinositol bisphosphate [13]. For instance, the cleavage products, inositol-triphosphate and diacylglycerol, activate the downstream signaling molecules protein kinase C and calcium-calmodulin kinase, respectively, which results in neurite outgrowth [21–23]. To confirm whether PLC involves in HNK-1-expressing FNx7 signaling, a PLC inhibitor, U73122, was used. U73122 suppressed neurite outgrowth on FNx7 regardless of the HNK-1 epitope, resulting in lengths similar to those following growth on PDL alone (**S3 Fig**), suggesting the possibility that the PLC signaling pathway may also be involved in HNK-1-induced neurite outgrowth.

CNTN is a GPI-anchored protein that expresses the HNK-1 epitope [24], although the function of the HNK-1 epitope on this protein has not yet been elucidated. Because the HNK-1 epitope on TNC in the extracellular matrix is important for neurite outgrowth, we hypothesized that the HNK-1 epitope on neuronal CNTN also participates in the neurite-promoting activity. To test this hypothesis, GlcAT-P and GlcAT-S-double gene knockout (WKO) neurons, which lack the HNK-1 epitope, were used to evaluate neurite length. When cultured on non-HNK-1 FNx7, WKO neurons showed significant neurite outgrowth (**S4A Fig**), which indicates that FNx7 is functional in WKO neurons. Although HNK-1-expressing FNx7 also exhibited neurite-promoting activity in WKO neurons, no significant increase in neurite length was observed with the HNK-1 epitope. Both wild-type (WT) and WKO brains expressed CNTN at similar levels at P0 (**S4B Fig**, upper). The HNK-1 epitope on CNTN was observed at an early stage but gradually decreased during development of the WT brain, whereas WKO brains showed no expression of the HNK-1 epitope on CNTN at P0 (**S4B Fig**, middle). These results suggest that the HNK-1 epitope on CNTN may regulate neurite outgrowth derived from HNK-1-expressing TNC.

## Discussion

**Fig 6:**
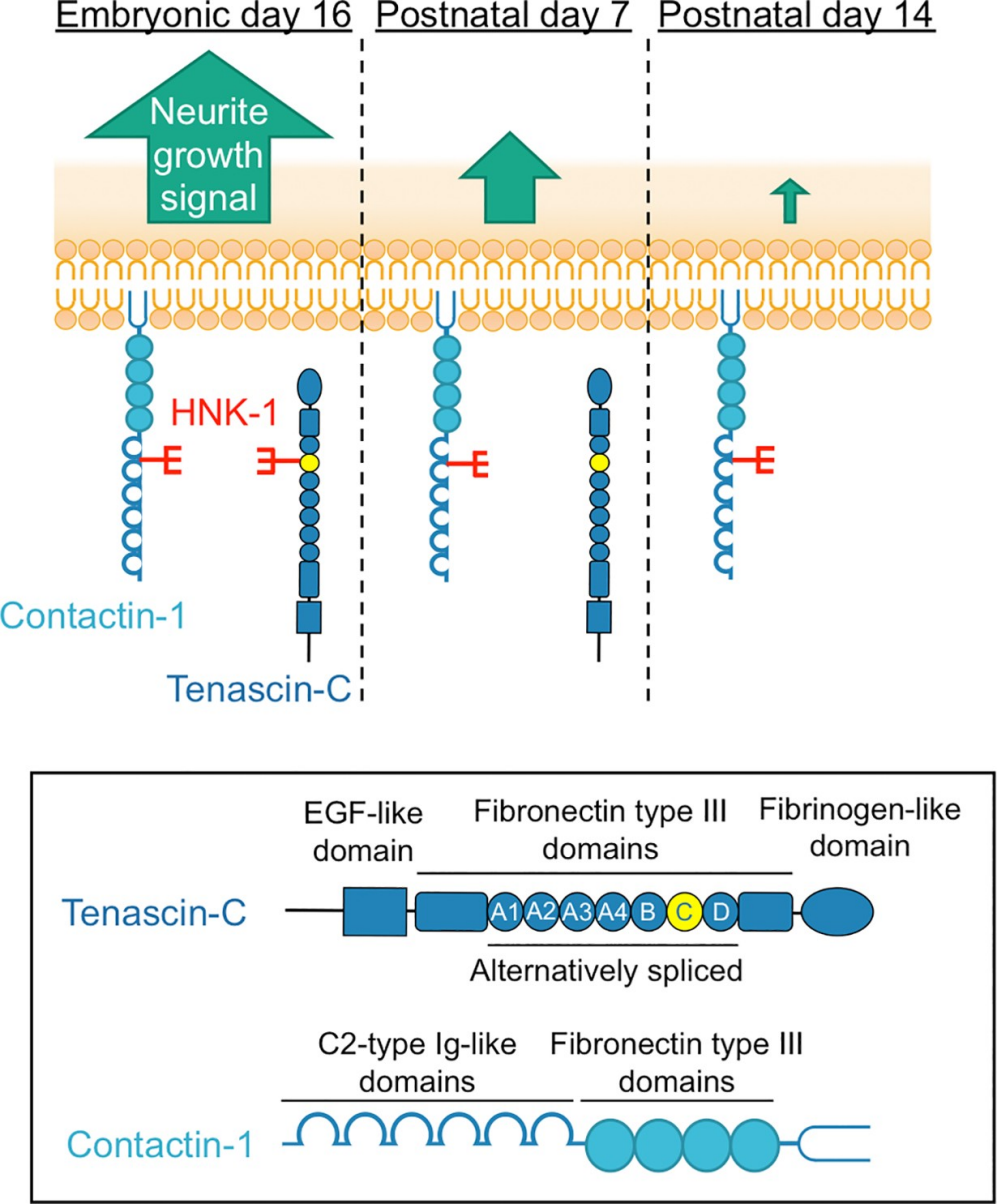
Model of neurite regulation by the HNK-1 epitope on tenascin-C and contactin-1. In E16 mouse brains, the HNK-1 epitope is expressed on both TNC and CNTN, inducing maximal neurite outgrowth, which could promote brain development and/or neuronal circuit formation (left panel). As development progresses, the HNK-1 epitope on TNC is nearly lost completely in the P7 mouse brain, whereas the epitope is expressed by CNTN. However, TNC is still expressed in the brain, and exhibits neurite-promoting activity, which might induce efficient neurite outgrowth via HNK-1-expressing CNTN in early development (middle panel). However, in the P14 mouse brain, TNC expression is almost abolished, resulting in weak neurite promotion (right panel).

We found that the HNK-1 epitope was primarily expressed on TNC in the mouse embryonic brain (**Fig 1**), which suggests that the HNK-1 epitope is involved in neuronal regulation in the embryonic brain by modulating TNC function. The HNK-1 epitope on TNC appears to regulate the proliferation of neural stem cells via modulation of epidermal growth factor receptor expression, as depletion of HNK-1 expression using small interfering RNA against HNK-1ST fails to induce the proliferation response [14]. TNC also possesses strong neurite-promoting activity [13,17–19]. Here, we investigated the effects of the HNK-1 epitope on the neurite-promoting activity of TNC. Of note, the largest isoform containing alternatively spliced FNIII repeats appeared to be modified with the HNK-1 epitope (**Fig 1**; [14]), which suggests that the HNK-1 epitope in the alternatively spliced FNIII repeats may be involved in neurite-promoting activity. Here, we demonstrated that the site-specific HNK-1 epitope on the C domain enhanced the neurite activity of FNx7 (**Figs 2–4**). Previous report showed that the substrate comprised of the C and D domains (CD) or the D and 6 domains (D6; 6 domain is one of the Fibronectin type III domains next to D domain) could elongate neurite length of hippocampal neurons, which is the similar extent to that of the B and D domains (BD) [18]. Lacking both the B and D domains did not show any significant neurite outgrowth. However, only BD could interact with CNTN, while CD and D6 could weakly associate with it [13,18], indicating that B domain is indispensable for the interaction with CNTN as well as neurite outgrowth. Here, we showed that the substrate lacking the D domain (FNx6) was enough to promote neurite outgrowth (the neurite length of FNx6 was comparable to that of FNx7; **Figs 2 and 3**). Furthermore, HNK-1 epitope enabled to enhance neurite outgrowth of FNx6 (**Fig 3**). Taken together, it is suggested that HNK-1 epitope on the C domain mainly regulates the function of the B domain. CNTN, a functional receptor of TNC, appeared to be required for the neurite-promoting activity of the HNK-1 epitope on TNC (**Fig 5**), which suggests that the HNK-1 epitope might enhance the binding of TNC to CNTN for downstream signaling. As the PLC inhibitor, U73122, suppressed the HNK-1 induced neurite outgrowth (**S3 Fig**), PLC might be involved in the downstream signaling. However, it is well-known that the PLC pathway involved in the neurite outgrowth is mediated not only by CNTN but also by the other neuronal receptors. Therefore, in order to address the precise signaling pathway derived from HNK-1 epitope on TNC, the further study will be required. Moreover, the presence of the HNK-1 epitope on extracellular TNC as well as neuronal CNTN may be involved in the same signaling pathway (**S4 Fig**). Overall, we propose the following model of neurite regulation by TNC and CNTN (**Fig 6**). HNK-1 expression on both TNC and CNTN results in maximal elongation of neurite length at E16. Expression of the HNK-1 epitope on TNC is decreased at P7 (**Fig 1A**), which can still promote significant neurite outgrowth because of TNC activity; however, TNC expression is mostly diminished at P14, resulting in reduced neurite outgrowth. Together, our results suggest that the HNK-1 epitope is important for the neurite-promoting activity of TNC, and likely the activity of CNTN, in the developing brain.

The involvement of TNC in higher brain function has been suggested by several reports. For instance, the induction of LTP and treatment with kainic acid increased the expression of TNC in the hippocampus [25,26] and mice stimulated in an enriched environment show stronger expression of TNC in the cerebellum [27]. Nonetheless, TNC-deficient mice failed to show gross phenotypes [28,29]. Subsequent experiments showed several interesting abnormalities: electronic properties are altered in the hippocampus [30]; anxiety-like behavior was decreased and exploring activity was increased in novel environments [31,32]; spontaneous nocturnal activity was increased [33]; improvement of sensorimotor learning in enriched environments was disabled [33]; and swimming ability and muscle strength were impaired [31,33]. These observations clearly suggest the physiological importance of TNC, although the precise underlying molecular mechanisms are unclear. The results of this study suggest that the spatiotemporal expression of the HNK-1 epitope on TNC and CNTN might cooperatively modulate brain development. This suggestion is also supported by the recent report, which revealed that the HNK-1 epitope on TNC facilitates the proliferation of mouse embryonic neural stem cells [14]. Taken together, inclusion of glycosylation in TNC function may be necessary for a comprehensive understanding of the molecular mechanisms underlying development of the embryonic brain.

Astrocyte is one of the sources for TNC in brain [34,35]. During the first three weeks of postnatal development, the glial cell population, which contains predominantly astrocytes, expands 6–8-fold in the rodent brain [36]. In contrast, TNC expression is dramatically suppressed during the period (**Fig 1A**; [37]). These results suggest that TNC expression is suppressed in astrocytes under the normal conditions. However, up-regulation of TNC in astrocyte is observed under some pathological conditions [38].

CNTN is also known as a characteristic neuronal receptor involved in axonal regulation [39]. A transgenic mouse model of CNTN showed enhanced spatial and object recognition memory with increased LTP in the CA1 region of the hippocampus [40]. In CNTN-deficient mice, misoriented parallel fibers in the cerebellum and fasciculation of granule cell axons were observed, which indicates that cerebellar organization is regulated by CNTN [41]. In addition, binding partners of CNTN on the neuronal membrane have also been investigated. For instance, the chondroitin sulfate proteoglycan RPTPβ/phosphacan interacts with CNTN in a heterophilic manner and induces neurite outgrowth [42]. Interestingly, CNTN receives a signal for neurite outgrowth from an extracellular source containing a specific chondroitin sulfate unit, called CS-E [GlcUA-GalNAc (4,6-O-disulfate)], which indicates that CNTN functions as a glycan-receptor [43]. The enhanced neurite-promoting activity of TNC with the HNK-1 epitope could be elicited by interaction with CNTN, because the HNK-1 epitope is a sulfated glycan similar to CS-E. Although the B domain of TNC reportedly interacts with CNTN [18], our data indicate that the HNK-1 epitope on the C domain is important for neurite outgrowth (**Fig 4**). This evidence suggests that interaction of the HNK-1 epitope on the C domain with CNTN enhances binding of the B domain to CNTN. However, further experiments are required to understand the relevance of the HNK-1 epitope on TNC to CNTN.

Our findings suggest that the HNK-1 epitope may support neuronal circuit formation in the developing brain. GlcAT-P-deficient mouse brains show normal morphology and gross anatomical features [5]. However, learning and memory formation are aberrant in adult GlcAT-P-deficient mice, which could be electrophysiologically explained by reduced LTP in the CA1 region of the hippocampus [5]. Abnormal morphology of dendritic spines of hippocampal neurons was observed in 2W GlcAT-P-deficient neurons but not in 10W GlcAT-P-deficient neurons, which suggests that the morphological abnormality of the dendritic spines was not the only cause of the phenotypes observed in adult GlcAT-P-deficient mice [44]. These phenotypes are supported by our model in **Fig 6**; the absence of the HNK-1 epitope on TNC and/or CNTN may induce subtle but crucial abnormalities in neuronal circuit formations. However, to understand the molecular association between the HNK-1 epitope and higher brain function, further research studies are required.

In summary, we found that the HNK-1 epitope on TNC enhances the neurite-promoting activity of TNC, and that this signaling pathway is mediated by CNTN, which may be regulated by HNK-1 epitope expressed on CNTN. These results strongly suggest that HNK-1 expression on TNC and CNTN coordinately regulates normal neuronal formation in the embryo. These findings further our understanding of the molecular mechanisms underlying the glycan-specific function of functional glycoproteins in the embryonic brain.

## Supporting information

S1 Fig Construction and expression of the recombinant alternatively spliced FNIII repeats. (A) Construction of each recombinant alternatively spliced FNIII repeat, composed of a combination of A1, A2, A3, A4, B, C, and D domains. Asterisks indicate the potential *N*-glycosylation sites and the number of sites is indicated. (B) Purified proteins with the HNK-1 epitope were immunoblotted using anti-myc pAb and HNK-1 mAb. Left and right blots were representative of 6 and 4 images of western blots, respectively. 0.2 μg proteins were loaded to each lane. (C) Each recombinant proteins pull down with Ni-NTA agarose beads were incubated with (+) or without (-) PNGase F and immunoblotted using anti-myc pAb and HNK-1 mAb. Each blot was representative of 4 images of western blots.
(TIF)

S2 Fig Treatment of FNx7 with or without HNK-1 into the culture medium did not show any neurite-promoting activity. Primary hippocampal neurons were cultured on PDL with treatment of FNx7 with or without HNK-1 into the culture medium for 3 days. Note that high concentrations (10 μg/ml; right panel) of the FNx7 also did not present the neurite-promoting activity. For 5 μg/ml concentrations, numbers of neurons measured without treatment, with FNx7 HNK-1(-), and with FNx7 HNK-1(+) were 88, 83, 56, respectively. For 10 μg/ml concentrations, numbers of neurons measured without treatment, with FNx7 HNK-1(-), and with FNx7 HNK-1(+) were 85, 81, 53, respectively. This result was obtained from a single culture. Error bars represent SEM. n.s., p > 0.05
(TIF)

S3 Fig Phopholipase C signaling pathway may involve in neurite outgrowth derived from the HNK-1 epitope on tenascin-C. The PLC inhibitor U73122 was added to the culture medium at a final concentration of 1 nM after 18 h of culture. Dark green, PDL treated with DMSO (control; n = 108); green, PDL treated with U73122 (n = 98); yellow, FNx7 HNK-1(-) treated with U73122 (n = 149); and orange, FNx7 HNK-1(+) treated with U73122 (n = 95). Numbers of measured neurons were indicated in parentheses. This experiment was performed across two independent cultures, respectively, and the representative result obtained from a single culture was shown. Error bars represent SEM. n.s., p > 0.05
(TIF)

S4 Fig HNK-1 epitope on contactin-1 may be required for HNK-1-expressing tenasicn-C-derived neurite outgrowth. (A) Primary hippocampal neurons prepared from GlcAT-P and GlcAT-S-double gene knockout (WKO) mouse brains were cultured on PDL, PDL coated with FNx7 without HNK-1 expression (HNK-1(-)), or PDL coated with FNx7 with HNK-1 expression (HNK-1(+)). Green, PDL; yellow, FNx7 HNK-1(-); and orange, FNx7 HNK-1(+). Numbers of neurons measured on PDL, FNx7 HNK-1(-), and FNx7 HNK-1(+) were 72, 132, 118, respectively. This experiment was performed across three independent cultures, and the representative result obtained from a single culture was shown. Error bars represent SEM. ***p < 0.001; **p < 0.01; n.s., p > 0.05. (B) Soluble fractions of wild-type or WKO brains prepared from E16, P0, P7, 2W, and 10W mouse brains were immunoblotted using anti-CNTN pAb, HNK-1 mAb, and anti-Tubulin mAb (for loading control). Each blot was representative of 2 images of western blots. 15, 50, and 20 μg proteins were loaded to each lane for CNTN, HNK-1, and Tubulin, respectively.
(TIF)

S5 Fig The images of the western blot membrane or CBB-stained gel including molecular size marker for Figs 1, 2 and 4. Dashed lines indicated the image presented in Figures. White or black arrows indicated the lanes of molecular size marker.
(TIF)

S6 Fig The images of the western blot membrane including molecular size marker for Supplementary Figs 1 and 4. Dashed lines indicated the image presented in Figures. White arrows indicated the lanes of molecular size marker.
(TIF)

S1 Table Minimal data set. (PDF)
