# Supplementary figures and images for: Site-specific HNK-1 epitope on alternatively spliced fibronectin type-III repeats in tenascin-C promotes neurite outgrowth of hippocampal neurons through contactin-1
Source: PLoS One. 2019 Jan 10;14(1):e0210193. doi: 10.1371/journal.pone.0210193 (PMC6328190; doi:10.1371/journal.pone.0210193)

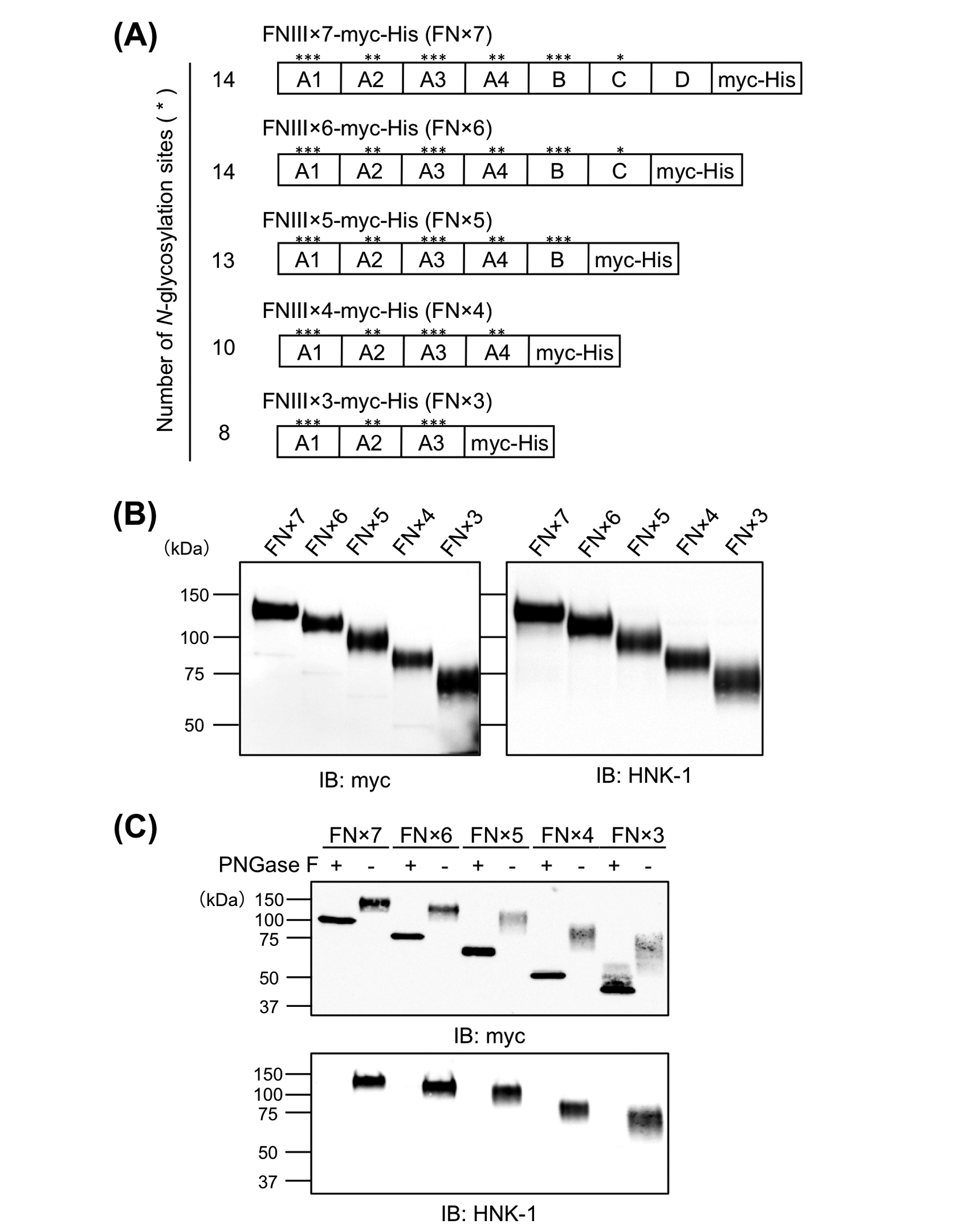

Supplement: S1 Fig — (A) Construction of each recombinant alternatively spliced FNIII repeat, composed of a combination of A1, A2, A3, A4, B, C, and D domains. Asterisks indicate the potential N-glycosylation sites and the number of sites is indicated. (B) Purified proteins with the HNK-1 epitope were immunoblotted using anti-myc pAb and HNK-1 mAb. Left and right blots were representative of 6 and 4 images of western blots, respectively. 0.2 μg proteins were loaded to each lane. (C) Each recombinant proteins pull down with Ni-NTA agarose beads were incubated with (+) or without (-) PNGase F and immunoblotted using anti-myc pAb and HNK-1 mAb. Each blot was representative of 4 images of western blots. (TIF) [file pone.0210193.s001.tif]

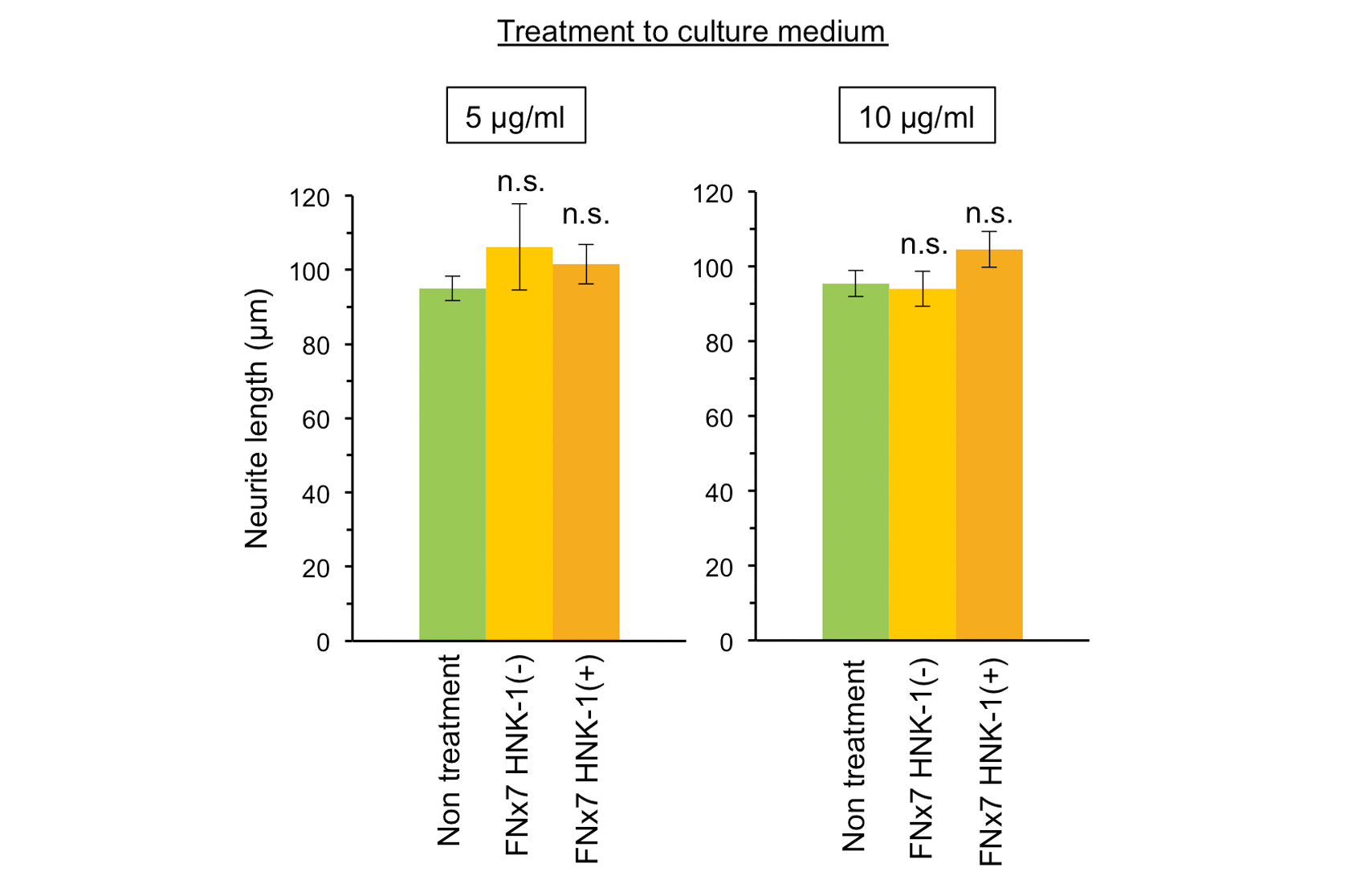

Supplement: S2 Fig — Primary hippocampal neurons were cultured on PDL with treatment of FNx7 with or without HNK-1 into the culture medium for 3 days. Note that high concentrations (10 μg/ml; right panel) of the FNx7 also did not present the neurite-promoting activity. For 5 μg/ml concentrations, numbers of neurons measured without treatment, with FNx7 HNK-1(-), and with FNx7 HNK-1(+) were 88, 83, 56, respectively. For 10 μg/ml concentrations, numbers of neurons measured without treatment, with FNx7 HNK-1(-), and with FNx7 HNK-1(+) were 85, 81, 53, respectively. This result was obtained from a single culture. Error bars represent SEM. n.s., p > 0.05 (TIF) [file pone.0210193.s002.tif]

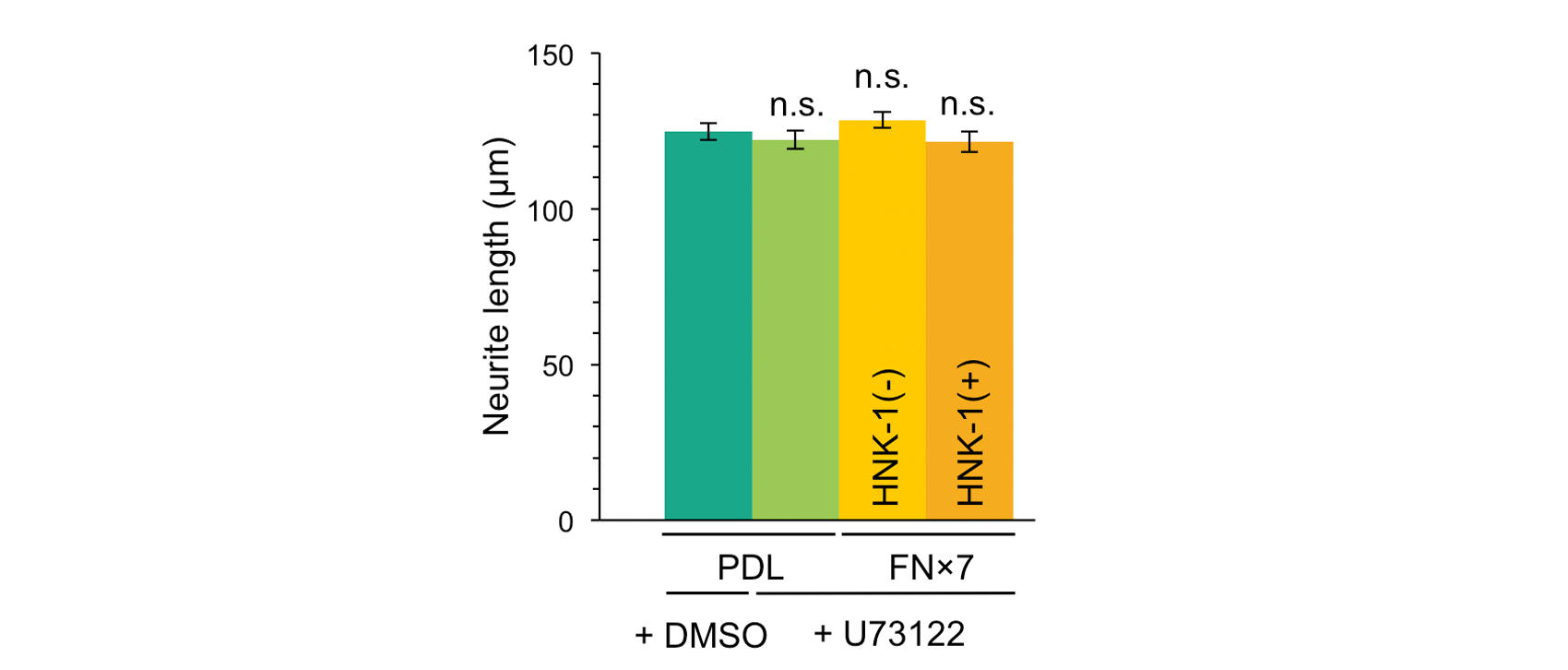

Supplement: S3 Fig — The PLC inhibitor U73122 was added to the culture medium at a final concentration of 1 nM after 18 h of culture. Dark green, PDL treated with DMSO (control; n = 108); green, PDL treated with U73122 (n = 98); yellow, FNx7 HNK-1(-) treated with U73122 (n = 149); and orange, FNx7 HNK-1(+) treated with U73122 (n = 95). Numbers of measured neurons were indicated in parentheses. This experiment was performed across two independent cultures, respectively, and the representative result obtained from a single culture was shown. Error bars represent SEM. n.s., p > 0.05 (TIF) [file pone.0210193.s003.tif]

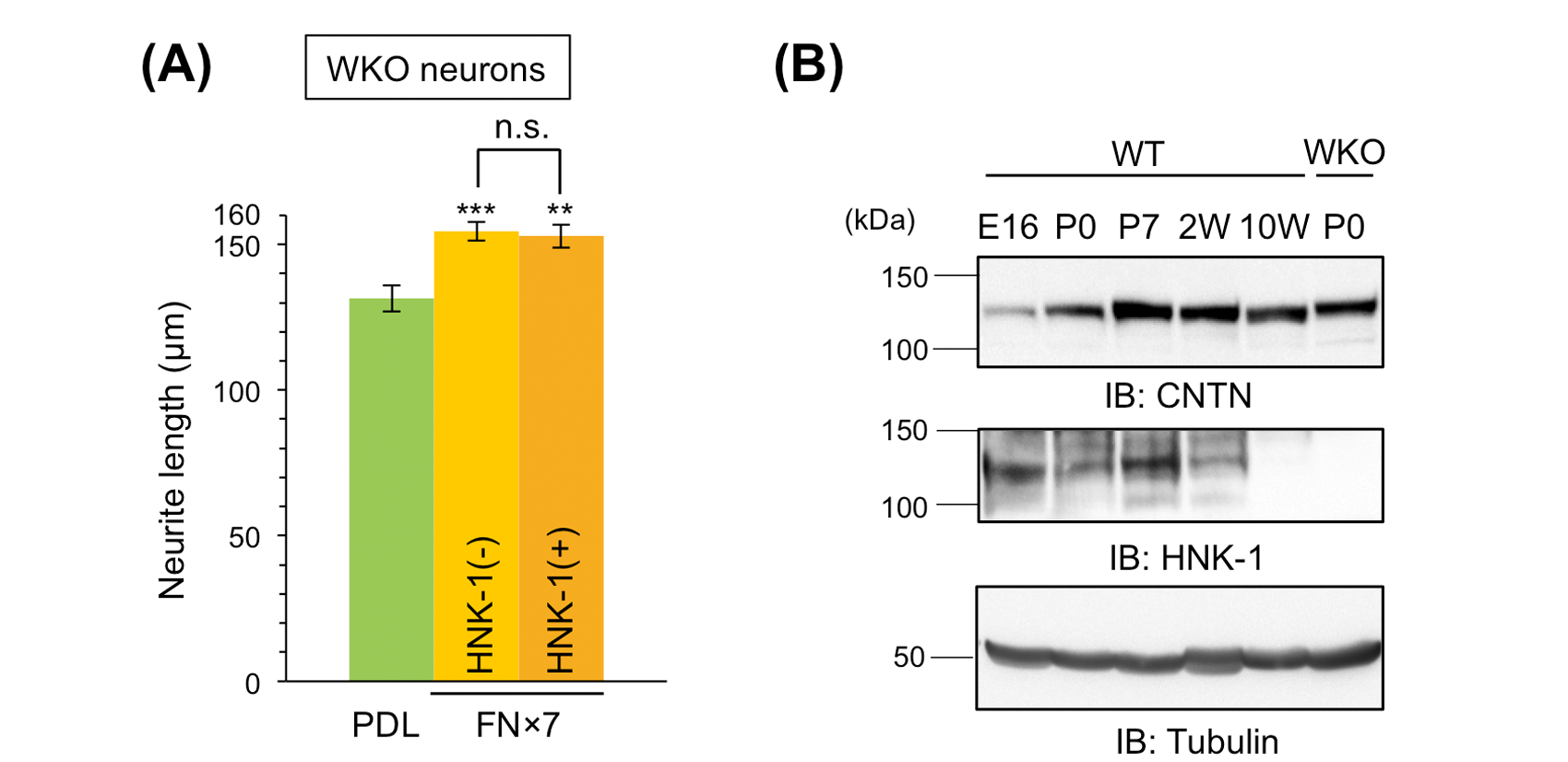

Supplement: S4 Fig — (A) Primary hippocampal neurons prepared from GlcAT-P and GlcAT-S-double gene knockout (WKO) mouse brains were cultured on PDL, PDL coated with FNx7 without HNK-1 expression (HNK-1(-)), or PDL coated with FNx7 with HNK-1 expression (HNK-1(+)). Green, PDL; yellow, FNx7 HNK-1(-); and orange, FNx7 HNK-1(+). Numbers of neurons measured on PDL, FNx7 HNK-1(-), and FNx7 HNK-1(+) were 72, 132, 118, respectively. This experiment was performed across three independent cultures, and the representative result obtained from a single culture was shown. Error bars represent SEM. ***p < 0.001; **p < 0.01; n.s., p > 0.05. (B) Soluble fractions of wild-type or WKO brains prepared from E16, P0, P7, 2W, and 10W mouse brains were immunoblotted using anti-CNTN pAb, HNK-1 mAb, and anti-Tubulin mAb (for loading control). Each blot was representative of 2 images of western blots. 15, 50, and 20 μg proteins were loaded to each lane for CNTN, HNK-1, and Tubulin, respectively. (TIF) [file pone.0210193.s004.tif]

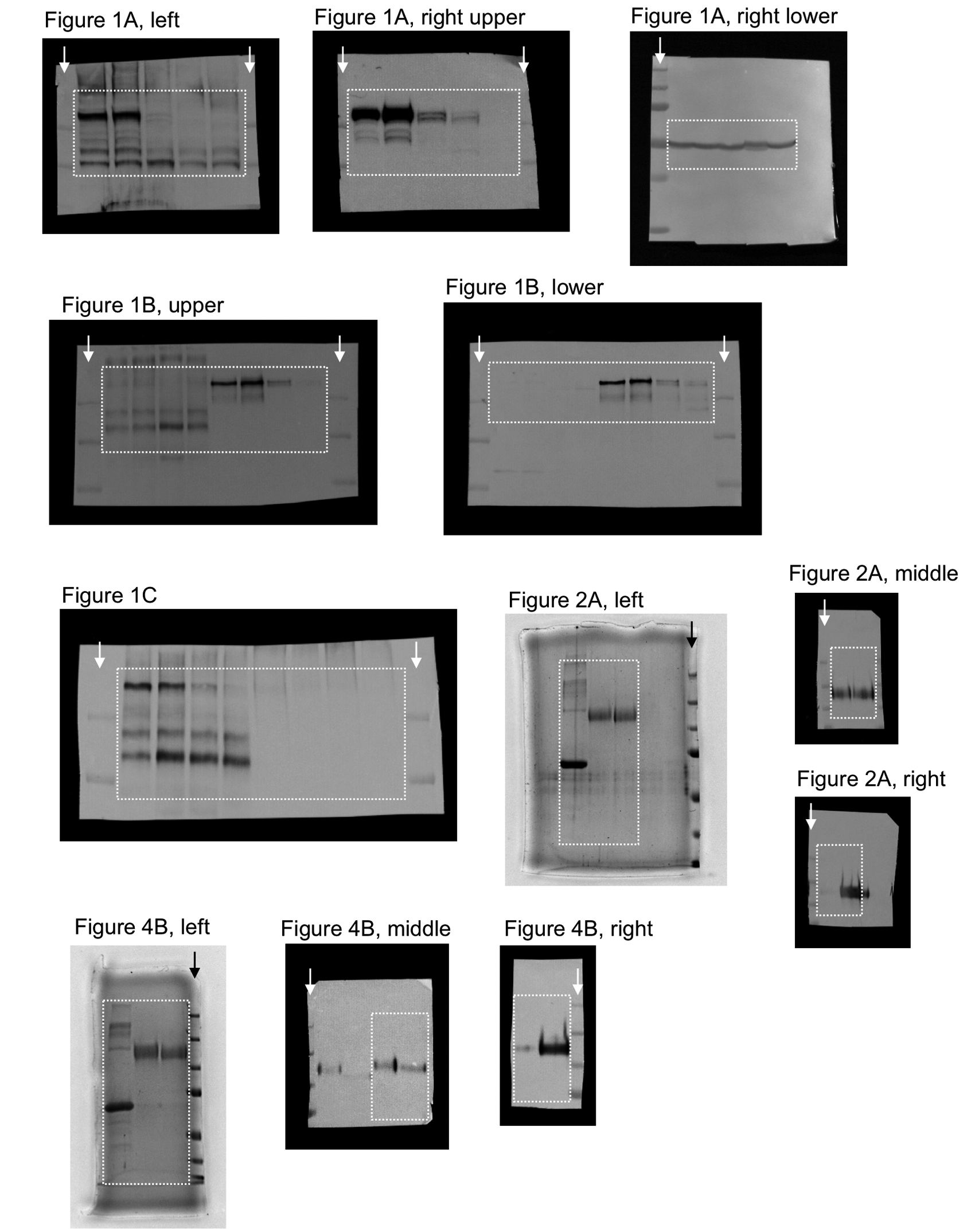

Supplement: S5 Fig — Dashed lines indicated the image presented in Figures. White or black arrows indicated the lanes of molecular size marker. (TIF) [file pone.0210193.s005.tif]

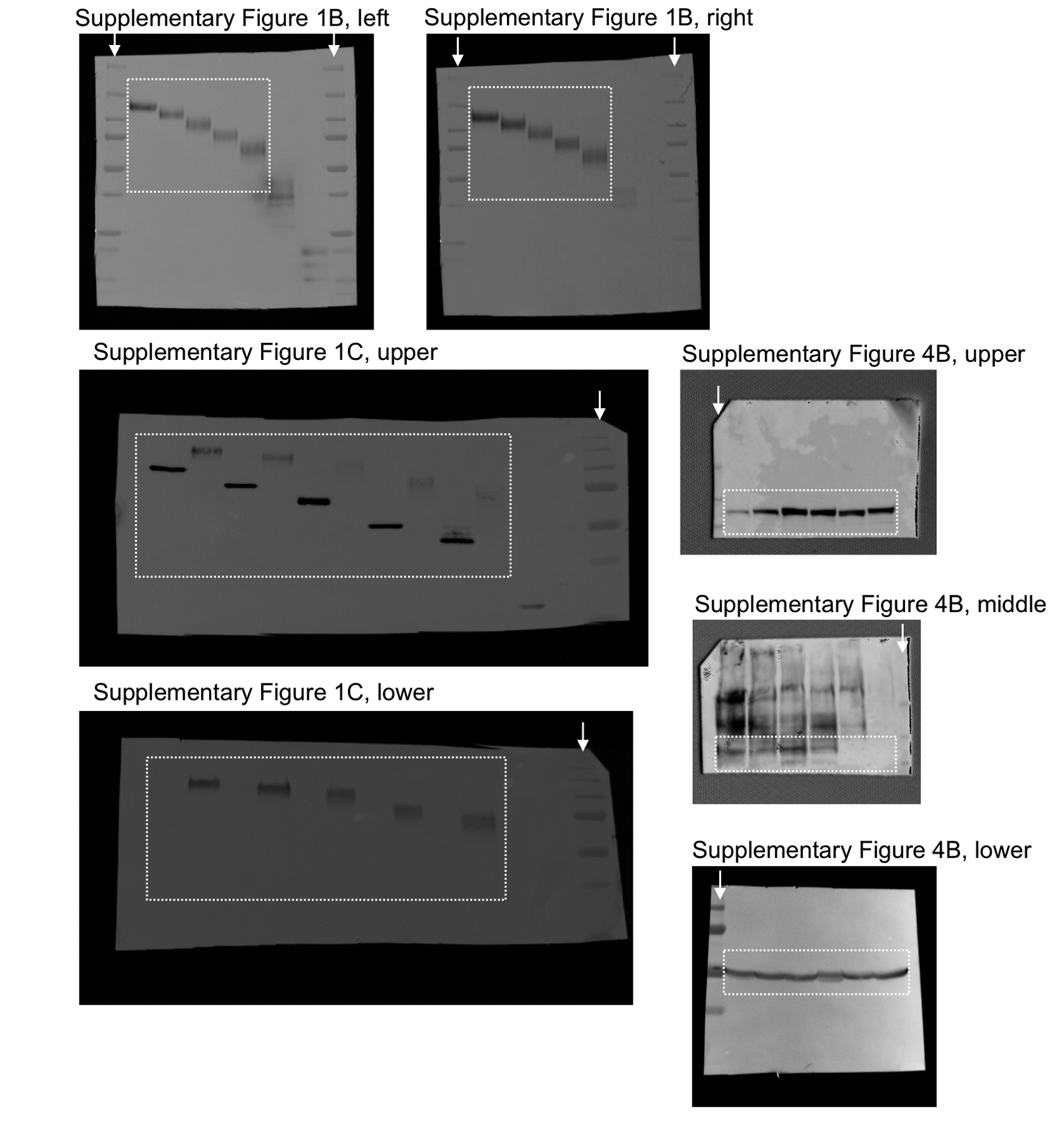

Supplement: S6 Fig — Dashed lines indicated the image presented in Figures. White arrows indicated the lanes of molecular size marker. (TIF) [file pone.0210193.s006.tif]
