# Supplementary material for: Site-specific HNK-1 epitope on alternatively spliced fibronectin type-III repeats in tenascin-C promotes neurite outgrowth of hippocampal neurons through contactin-1
Source: PLoS One. 2019 Jan 10;14(1):e0210193. doi: 10.1371/journal.pone.0210193 (PMC6328190; doi:10.1371/journal.pone.0210193)
Supplement: S1 Table — (PDF) [file pone.0210193.s007.pdf]

Fig. #	Mean	SD	SEM	Statistical method used	P value	n
Fig. 2C						
PDL	107.40	21.74	2.74	One-way ANOVA	***p<0.001	63
FNx7 HNK-1(-)	125.98	31.17	2.65	with Tukey's multiple		138
FNx7 HNK-1(+)	144.61	6.56	3.81	comparison posttest		43
Fig. 3A						
PDL	111.90	18.24	1.60	One-way ANOVA	***p<0.001	130
FNx6 HNK-1(-)	133.45	24.97	1.97	with Tukey's multiple		161
FNx6 HNK-1(+)	145.12	30.98	2.51	comparison posttest		152
Fig. 3B						
PDL	109.13	25.51	2.23	One-way ANOVA	n.s. p>0.05	131
FNx5 HNK-1(-)	124.26	22.54	1.45	with Tukey's multiple	***p<0.001	241
FNx5 HNK-1(+)	128.43	29.70	2.48	comparison posttest		143
Fig. 3C						
PDL	111.50	36.21	3.90	One-way ANOVA	n.s. p>0.05	86
FNx3 HNK-1(-)	108.72	26.24	2.36	with Tukey's multiple		124
FNx3 HNK-1(+)	108.92	24.47	2.55	comparison posttest		92
Fig. 4C						
PDL	108.83	21.64	1.60	One-way ANOVA	n.s. p>0.05	184
N1534Q HNK-1(-)	122.98	32.06	2.28	with Tukey's multiple	***p<0.001	197
N1534Q HNK-1(+)	124.67	24.60	1.76	comparison posttest		195
Fig. 5						
PDL + normal IgG	116.52	24.69	1.67	One-way ANOVA	n.s. p>0.05	219
PDL + CNTN Ab	118.53	28.81	2.53	with Tukey's multiple	***p<0.001	130
FNx7 HNK-1(-) + normal IgG	134.21	26.87	2.28	comparison posttest		139
FNx7 HNK-1(-) + CNTN Ab	116.55	19.38	1.52			163
FNx7 HNK-1(+) + normal IgG	142.33	32.86	2.65			154
FNx7 HNK-1(+) + CNTN Ab	119.63	25.79	2.00			166

S2 Fig.						
Non treatment	94.99	31.48	3.36	One-way ANOVA with Tukey’s multiple comparison posttest	n.s. $p>0.05$	88
FNx7 HNK-1(-) 5 µg/ml	106.15	44.79	11.65			83
FNx7 HNK-1(+) 5 µg/ml	101.56	39.98	5.34			56
Non treatment	95.46	32.08	3.48			85
FNx7 HNK-1(-) 10 µg/ml	94.07	42.04	4.67			81
FNx7 HNK-1(+) 10 µg/ml	104.63	35.01	4.81			53
S3 Fig.						
PDL with DMSO	124.78	28.72	2.76	One-way ANOVA with Tukey’s multiple comparison posttest	n.s. $p>0.05$	108
PDL with U73122	122.13	29.34	2.96			98
FNx7 HNK-1(-) with U73122	128.43	32.28	2.64			149
FNx7 HNK-1(+) with U73122	121.38	31.34	3.22			95
S4A Fig.						
PDL	131.46	38.46	4.53	One-way ANOVA with Tukey’s multiple comparison posttest	n.s. $p>0.05$ **$p<0.01$ ***$p<0.001$	72
FNx7 HNK-1(-)	154.52	37.56	3.27			132
FNx7 HNK-1(+)	152.79	42.62	3.92			118
